# Verification of Continuum Mechanics Predictions with Experimental Mechanics

**DOI:** 10.3390/ma13010077

**Published:** 2019-12-22

**Authors:** Cesar A. Sciammarella, Luciano Lamberti, Federico M. Sciammarella

**Affiliations:** 1 Department of Mechanical, Materials and Aerospace Engineering, Illinois Institute of Technology, Chicago, IL 60616, USA; sciammarella@iit.edu; 2 Dipartimento di Meccanica, Matematica e Management, Politecnico di Bari, 70126 Bari, Italy; 3 MXD Corporation, 1415 N. Cherry Avenue, Chicago, IL 60642, USA

**Keywords:** representative volume element (RVE), kinematical variables, derivatives of displacements, large deformations, constitutive models, Al–SiC composite material, urethane rubber

## Abstract

The general goal of the study is to connect theoretical predictions of continuum mechanics with actual experimental observations that support these predictions. The representative volume element (RVE) bridges the theoretical concept of continuum with the actual discontinuous structure of matter. This paper presents an experimental verification of the RVE concept. Foundations of continuum kinematics as well as mathematical functions relating displacement vectorial fields to the recording of these fields by a light sensor in the form of gray-level scalar fields are reviewed. The Eulerian derivative field tensors are related to the deformation of the continuum: the Euler–Almansi tensor is extracted, and its properties are discussed. The compatibility between the Euler–Almansi tensor and the Cauchy stress tensor is analyzed. In order to verify the concept of the RVE, a multiscale analysis of an Al–SiC composite material is carried out. Furthermore, it is proven that the Euler–Almansi strain tensor and the Cauchy stress tensor are conjugate in the Hill–Mandel sense by solving an identification problem of the constitutive model of urethane rubber.

## 1. Introduction

**Figure 1:**
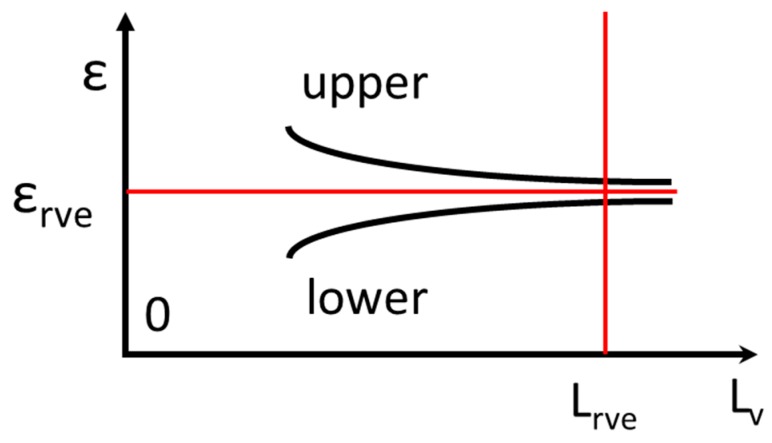
Variation of local strain value with respect to the representative volume element scale.

The continuum mechanics hypothesis relies on the concepts of representative volume element (RVE) [1,2,3] and statistical volume element (SVE) [4]. The RVE relates probabilistic outcomes based on the theory of stochastic variables with a deterministic outcome based on physical laws. The idea of RVE can be explained as follows. One determines a given value of strain ε developed in a certain volume of a solid medium. This value is related to a certain volume of material with a characteristic dimension L. However, the volume includes microcomponents of characteristic size d. The ratio δ = L/d is referred to as the mesoscale. For a given δ, responses ε may change for different materials, each of which has a different “d”. Figure 1 shows that for a given L, as δ changes, responses of different volumes converge to a certain value ε_rve_ (the subscript “rve” corresponds to RVE). The scatter of different values disappears, and a deterministic value can be reached within a certain number of significant figures. This behavior, called ergodicity, corresponds to statistical analysis of random variables. Ergodicity implies that for time-independent processes, the spatial average ε_rve_ is equal to the ensemble average. Hence, if strains are measured within the RVE size order of magnitude, their average converges to a single value of ε_rve_. The same should occur for any RVE of the analyzed material. The selected RVE size allows to capture the local value of the considered variable. The local values must change marginally if the representative volume slightly deviates from some optimum value. This concept is clarified by Figure 1. As the characteristic length reaches the L_rve_ value, ε_rve_ takes an asymptotic value defined by a satisfactory number of significant figures.

The theoretical issues discussed above should be carefully considered when measurements are performed by means of optical techniques such as moiré, holography, speckle, and digital image correlation, which spatially sample the analyzed volumes. For methods utilizing carrier signals, sampled volumes depend on the pitch p of the tagged signals as well as on the modulating carrier that makes displacement determination feasible. In digital image correlation (DIC), the representative volume element depends on the size of sub-elements and the number of pixels included in each sub-element. This concept applies to kinematic properties such as strains as well as to force-based quantities such as stresses σ. The required conditions rely on the Hill–Mandel homogenization principle [5,6], which involves the space of admissible displacements in the RVE. Generally speaking, for σ_rve_ and ε_rve_, the virtual work in the macro scale equals the virtual work in the subscale. This condition must be considered when constitutive models are derived in order to select kinematic variables and corresponding dynamic variables that are mutually compatible.

This study presents an experimental verification of the RVE concept; theoretical concepts must be supplemented with experimental tools. To connect molecular dynamics with models derived from continuum mechanics is not an easy task. For this purpose, the moiré method [7,8], a typical tool used in experimental mechanics for measuring displacements, will be utilized. The aim is to apply tools available in novel developments introduced by the authors in different papers [9,10,11,12,13,14], putting together successive derivations in the field of the kinematics of the continuum. To help the readers, derivations extracted from the different publications will be provided. This analysis will be limited to 2D for the sake of simplicity. Isothetic lines (moiré fringes) encode the vectorial displacement field referring to the deformed configuration. This Eulerian representation of deformation is related to a differential geometry representation of the RVE. Hence, strains can be derived from a tensor representation of the displacement derivatives. Another important aspect is to relate kinematical and dynamical variables in order to satisfy compatibility conditions between a selected stress tensor and the compatible strain tensor. The paper provides an original derivation of the relationship between both tensors in the context of an RVE and an adopted constitutive function.

The moiré patterns recorded for an Al–SiC particulate composite material subject to tensile loading and an urethane rubber disk subject to diametrical compression are analyzed. In the first example, the information gathered at the microscale from the chosen RVE are matched with the information gathered at the macro scale on a 10,000 times larger region of the specimen; failure mechanisms derived from the experimentally obtained strain distribution are compared with finite element predictions. In the second example, the suitability of the constitutive model hypothesized for urethane rubber is assessed, also verifying the compatibility of the stress and strain tensors.

The experimental mechanics-based approach adopted in this study for verifying the RVE concept is very general, while the technical literature usually focuses on determining the proper homogenization scheme as well as on the RVE shape and size to be used in specific homogenization-based computations on heterogeneous materials such as, for example, composite materials reinforced by fibers, random fibers, textures, or randomly distributed inclusions [15,16,17,18,19,20]. Regarding the specific application of optical methods to RVE analysis, it should be mentioned that digital image correlation was recently used for multiscale investigation on woven composites [21,22]. Following the classical approach of the literature, the length scale of the representative volume element (RVE) was estimated by comparing different window sizes where the average of the local strain pattern measured by DIC has to match macro-scale measurements. The present study is some steps ahead of Refs. [21,22] because the concept of RVE will be framed in a very comprehensive context connecting image analysis, kinematics of deformation, the material’s structure, the constitutive behavior, and failure mechanisms. This must be done because while the woven composite analyzed in Refs. [21,22] had a fairly regular structure, the Al–SiC composite analyzed in this study has a highly heterogeneous structure where strain concentrations change by a great deal from one particle–matrix interface region to another.

The article is structured as follows. The local kinematical variables used in the analysis of moiré fringes are described in Section 2. The process of transforming recorded gray-level patterns into vectorial fields corresponding to displacement components is described in Section 3. A complex analysis of vectorial displacement fields is presented in Section 4, while derivatives of displacements with respect to Eulerian deformed coordinates, and the corresponding tensor notation are illustrated in Section 5. Section 6 describes deformation in terms of differential geometry with special emphasis on the Eulerian description and focusing on the effect of rigid body rotations. Section 7 deals with the relationships between kinematical variables and dynamical variables. Section 8 reviews the process of derivation of constitutive functions relating kinematics and dynamics variables. Section 9 is devoted to explaining the relationship among the concept of RVE and experimental mechanics measurements. Section 10 presents the experimental verification that the Euler–Almansi and the Cauchy tensors are conjugates of each other for hyperelastic materials. Section 11 summarizes the content of the paper and includes some concluding remarks.

With reference to notation, we consider real or complex-valued functions f(**x**) defined on **ℜ**_n_ or C, where n = 1, 2. Ordinary case letters represent scalar quantities, and bold letters represent vectorial quantities. It will be written f(**x**) or f(x, y), where the bold lowercase indicates a vector quantity or we will list the lowercase variables, whichever is more convenient in the context of the discussion.

## 2. Local Kinematic Variables

The considered issue in this section is the computation of local values of displacements and displacement derivatives in the presence of large deformations and large rotations. The conceptual basis and developments of the material included in this section and some of the following sections are presented in detail in [9,10,11,12,13,14].

In Refs. [13,14], introducing OSA (Optical Signal Analysis), a new method for fringe pattern analysis, the concept of RVE is connected to the definition of the local kinematic variables by the selection of the scale L, the pitch of the tagged carrier p, and the carrier fringes generated by adding a carrier via frequency shifting or other approaches.

**Figure 2:**
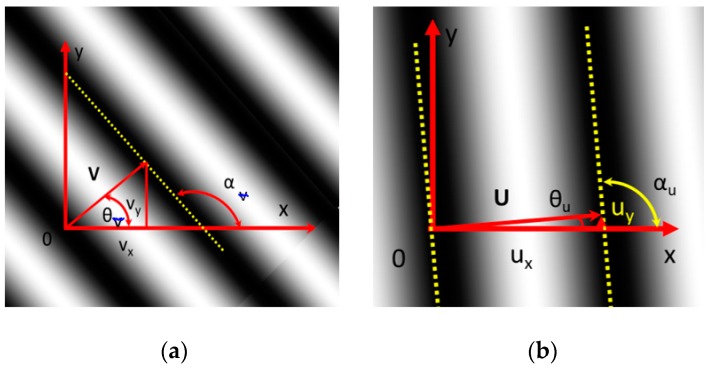
(**a**) Example of a local pattern of vertical displacement **V** (fringes in the undeformed condition are horizontal) obtained for large deformations and rotations; (**b**) Example of a local pattern of horizontal displacement **U** obtained for small deformations and rotations.

Figure 2a is a 2D version of the more general 3D diagrams of [12] and represents a local region (RVE) of vertical displacements, which satisfies the condition illustrated in Figure 1. The displacement vector **V**, which should be vertical, presents instead a significant horizontal component v_x_ because of the presence of a rigid body rotation. The angle α_v_ defining the direction of fringes should be α_v_ = 0 in case there is no rotation. However, its value measured counterclockwise with respect to the X-axis (see Figure 2a) is α_v_ > π/2. In Figure 2b, α_u_ for the **U** displacements should be α_u_ = π/2, but the actual angle is slightly larger than π/2. Since the angle θ_u_ is almost equal to zero, the modulus of **U** is approximately equal to u_x_ ≈ u, where u is the projection of the horizontal displacement along the x-axis; hence, the component u_y_ can be neglected.

**Figure 3:**
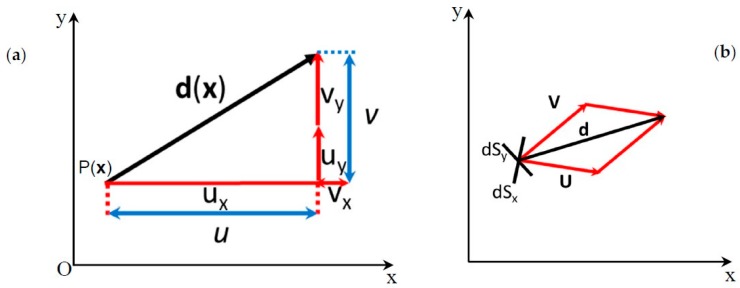
(**a**) Graphical representation of the displacement vector **d**(**x**) and its projected components *u* and *v*; (**b**) Vectorial sum of the two component vectors.

Figure 3 shows the displacement vector **d**(**x**) generated at a given point P(**x**) of the continuum. The position of the point P is defined by the vector **x**.

For large deformations and large rotations, the following relationship was derived in [13,14]

(1) $$\left\{ \begin{array}{l} d_{x}(x{)=u}_{x}\left(x\right)+v_{x}\left(x\right)=u(x)\\ d_{y}(x{)=u}_{y}\left(x\right)+v_{y}\left(x\right)=v(x) \end{array}\right.$$

which corresponds to the typical equations utilized for moiré fringes or isothetic lines.

## 3. Vectorial Fields of Recorded Gray Levels

This section explains how vectorial kinematical variables are obtained from scalar signals collected as gray levels in an image of an RVE.

The recorded images are scalar functions of gray levels that can be transformed into vectorial fields representing displacement components [13,14]. The recorded image is described by a 2D real scalar potential of gray levels F_2R_. Introducing carrier fringes and using Cartesian coordinates, the F_2R_ potential can be expressed as the sum of two scalar components:

F_2R_(**x**) = U(**x**) + V(**x**) (2)

The scalar functions U(**x**) and V(**x**) describe two systems of carrier fringes that can be converted into vectorial functions using the Fourier transform or the Hilbert transform [13,14]. From Figure 3b, it can be written:

**d**(**x**) = **U**(**x**) + **V**(**x**) (3)

Applying the gradient operation to Equation (2) and considering its distributive property, it follows:

∇F_2R_(**x**) = ∇U(**x**) + ∇V(**x**) (4)

The above operation leads to obtain the projection equations:

(5) $$\left\{ \begin{array}{l} \frac{\partial d_{x}(x)}{\partial x}=\frac{\partial u_{x}\left(x\right)}{\partial x}+\frac{\partial v_{x}\left(x\right)}{\partial x}=\frac{\partial u}{\partial x}\\ \frac{\partial d_{y}(x)}{\partial y}=\frac{\partial u_{y}\left(x\right)}{\partial y}+\frac{\partial v_{y}\left(x\right)}{\partial y}=\frac{\partial v}{\partial x} \end{array}\right..$$

**Figure 4:**
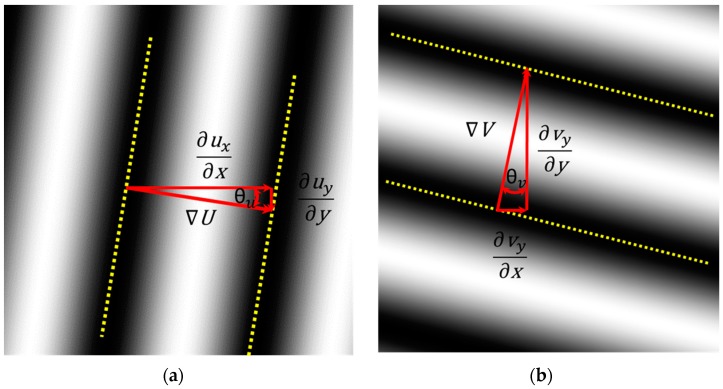
Schematic of local gradients vectors and corresponding Cartesian components for (**a**) U and (**b**) V projected displacements.

Figure 4 illustrates the derivative components defined by Equation (5). It can be seen that vectors **U**(**x**) and **V**(**x**) are co-axial with vectors ∇U(**x**) and ∇V(**x**). However, scales are changed.

Equation (4) can be rewritten as:

(6) $$\nabla F_{2R}=\frac{\partial F_{2R}}{\partial x}i+\frac{\partial F_{2R}}{\partial y}j.$$

Putting ∇F_2R_ = **G**_2_(***x***) and computing the divergence of the **G**_2_ vector, it follows:

(7) $$\nabla •G_{2}\left(x\right)=\frac{\partial^{2}F_{2R}}{\partial^{2}x}+\frac{\partial^{2}F_{2R}}{\partial^{2}y}.$$

The curl (i.e., the rotor) of the vector field **G**_2_(***x***) is defined as:

(8) $$\nabla \times G_{2}=\left(\frac{\partial^{2}F_{2R}\left(x\right)}{\partial y\partial x}-\frac{\partial^{2}F_{2R}\left(x\right)}{\partial x\partial y}\right)k.$$

If the displacement field is irrotational (i.e., it does not include any rotations), both sides of Equation (8) become equal to zero; that is:

(9) $$\nabla \times G_{2}=\left(\frac{\partial^{2}F_{2R}(x)}{\partial y\partial x}-\frac{\partial^{2}F_{2R}(x)}{\partial x\partial y}\right)k=0.$$

If the divergence and the curl of **G**_2_(***x***) are both equal to zero, the following relationships between the second-order derivatives of the potential F_2R_ can be written:

(10) $$\left\{ \begin{array}{l} \frac{\partial^{2}F_{2R}}{\partial^{2}x}=-\frac{\partial^{2}F_{2R}}{\partial^{2}y}\\ \frac{\partial^{2}F_{2R}}{\partial y\partial x}=\frac{\partial^{2}F_{2R}}{\partial x\partial y} \end{array}\right..$$

Replacing Equation (2) in Equation (10), it follows:

(11) $$\left\{ \begin{array}{l} \frac{\partial^{2}\left(U(x)+V(x)\right)}{\partial^{2}x}=-\frac{\partial^{2}\left(U(x)+V(x)\right)}{\partial^{2}y}\\ \frac{\partial^{2}\left(U(x)+V(x)\right)}{\partial y\partial x}=\frac{\partial^{2}\left(U(x)+V(x)\right)}{\partial x\partial y} \end{array}\right..$$

From Equations (10) and (11), it follows that the gray level’s scalar potential $F_{2R}(x)$ that defines the local phase must be a solution of the Laplace’s equation; that is:

(12) $$\frac{\partial^{2}F_{2R}(x)}{\partial^{2}x}+\frac{\partial^{2}F_{2R}(x)}{\partial^{2}y}=0.$$

The solutions of the Laplace’s equation are harmonic functions that follow the potentials theory. It should be noted that the present study deals with the concept of local values associated to the RVE. In order to utilize the adopted model of optical signals, it is necessary to have carrier fringes of higher frequency than those corresponding to signal local values [13,14]. Such a requirement is graphically expressed by Figure 2 and Figure 4, which show that local values of gray levels vary as harmonic functions.

When displacement fields are not irrotational and not conservative, the solution of the 2D continuum field is obtained by solving the Poisson’s equation:

(13) $$\frac{\partial^{2}F_{2R}(x)}{\partial^{2}x}+\frac{\partial^{2}F_{2R}(x)}{\partial^{2}y}=P_{s}(x).$$

where P_s_(**x**) is a source term. In summary, the partial differential Equations (12) and (13) provide solutions for any type of 2D displacement fields.

The above derivations are consistent with the continuum mechanics analytic solutions of 2D problems and allow displacement fields and their derivatives to be experimentally determined.

## 4. Complex Plane Analysis of Displacement Functions

This section provides the connection of scalar potential functions and their representation in the complex plane that makes it possible through the corresponding software to compute the necessary kinematic variables.

In the previous section, we have derived the mathematical relationships between scalar potential functions and displacement vectors for the recorded images defined by scalar gray levels. These potentials must be connected with the OSA method in order to obtain the actual displacement components from the recorded distributions of gray levels. For that purpose, operations must be moved from the physical space to the complex space. It was proven in [13,14] that scalar gray levels of the image correspond to a complex potential in the complex plane. This leads to some interesting considerations on the methods used for retrieving displacements from gray-level scalar fields.

Let us consider the complex plane C and define complex potentials $U_{C}^{e}(x_{c})$ and $V_{c}^{o}(x_{c})$. The upper scripts “e” and “o”, respectively, indicate that the potential can be decomposed into one even function and another odd function. One defines two complex potentials,

(14) $$\{\begin{array}{c} z_{xc}(x_{c})=U_{c}^{e}(x_{c})\overset{\Rightarrow }{i}+U_{c}^{0}(x_{c})\overset{\Rightarrow }{j}\\ z_{yc}(x_{c}{)=V}_{c}^{e}(x_{c})\overset{\Rightarrow }{i}+V_{c}^{o}(x_{c})\overset{\Rightarrow }{j} \end{array}$$

where the symbol ⇒ refers to the versors of the complex plane. If all the potential components are real and can be differentiated at a given point of the complex plane, the $U_{C}^{e}(x_{c})$ and $V_{c}^{o}(x_{c})$ functions must satisfy the Cauchy–Riemann conditions in the complex plane. Hence, it can be written:

(15) $$\left\{ \begin{array}{l} \frac{\partial U_{c}^{e}}{\partial x_{c}}=\frac{\partial U_{c}^{o}}{\partial y_{c}}\\ \frac{\partial U_{c}^{e}}{\partial y_{c}}=-\frac{\partial U_{c}^{o}}{\partial x_{c}} \end{array}\right.$$

Similar equations can be written for the other potential function corresponding to V. Equation (15) connects the Hilbert transform, holomorphic functions, and the gray levels expressed as potential functions that lead to defining a local phase. Hence, a consistent mathematical framework is adopted in order to support the OSA methodology of displacement retrieval utilizing the gray levels recorded in an image. For example, let us assume that $U_{c}^{o}(x)$ takes the form

(16) $$U_{xc}^{e}(x_{c})=I_{p}\cos\phi \left(x\right).$$

Taking the Hilbert transform of the above equation, we can write:

(17) $$U_{xc}^{o}(x_{c})=I_{q}\sin\phi \left(x\right).$$

It was proven in [14] that Equations (16) and (17) are approximately valid for optically recorded displacement fields. The $U_{C}^{e}(x_{c})$ and $V_{c}^{o}(x_{c})$ functions must be approximately stationary in the REV (see in Figure 1). The Poincare sphere [11] provides a graphical representation of the above-stated functions. This 3D plot in the complex space shows the necessary components that allow the state of deformation of a pixel in the 2D physical space to be experimentally determined.

## 5. Derivatives of Displacements

This section summarizes the properties of the derivatives of the displacements that are the main tools in the implementation of a measure of the deformation that complies with a fundamental property: the invariance of this measure with rigid body rotations in the RVE.

Derivatives of displacements represent a fundamental step in solving experimental mechanics problems. In [7] and the references given therein, procedures are given to get derivatives directly from fringe patterns without the need to previously obtain displacements. In [9], the authors reviewed the basic concepts entailed by the description of the deformation of a medium and the role of the displacement derivatives. This section summarizes the material of [9] and focuses on the Eulerian description. One condition that displacement derivatives must satisfy is that the Jacobian of the 2D coordinate change is not equal to zero [9]; that is:

(18) $$J_{2}=\det\left|\begin{array}{cc} \frac{\partial x}{\partial X} & \frac{\partial x}{\partial Y}\\ \frac{\partial y}{\partial X} & \frac{\partial y}{\partial Y} \end{array}\right|\neq 0.$$

where (x, y) are the Eulerian coordinates and (X, Y) are the Lagrangian coordinates of a point of the 2D continuum currently analyzed. The displacement vector of a point has two components: (i) relative change of distance between the point and a neighbor point; and (ii) rigid body displacements caused by the deformation of the body region hosting the analyzed point. The 2D displacement vector is consistently defined with Equation (1):

(19) $$\{\begin{array}{c} d_{x}=u\\ d_{y}=v \end{array}.$$

Finally, the tensor containing the derivatives of displacements in the Eulerian description is defined [9]:

(20) $$[J]=\left[\begin{array}{cc} \frac{\partial u}{\partial x} & \frac{\partial u}{\partial y}\\ \frac{\partial v}{\partial x} & \frac{\partial v}{\partial y} \end{array}\right].$$

The trace of this tensor corresponds to the linear strain invariant in the Eulerian description. The [J] tensor can be decomposed into a symmetric part and an anti-symmetric part. The symmetric part of the [J] tensor is defined as:

(21) $$\left[J_{s}\right]=\left[\begin{array}{cc} \frac{\partial u}{\partial x} & \frac{1}{2}\left(\frac{\partial u}{\partial y}+\frac{\partial v}{\partial x}\right)\\ \frac{1}{2}\left(\frac{\partial v}{\partial x}+\frac{\partial u}{\partial y}\right) & \frac{\partial v}{\partial y} \end{array}\right].$$

The anti-symmetric part of the [J] tensor is defined as:

(22) $$\left[J_{a}\right]=\left[\begin{array}{cc} 0 & \frac{1}{2}\left(\frac{\partial u}{\partial y}-\frac{\partial v}{\partial x}\right)\\ \frac{1}{2}\left(\frac{\partial v}{\partial x}-\frac{\partial u}{\partial y}\right) & 0 \end{array}\right].$$

The tensor [J*_s_*] contains the derivatives related to the deformation components of the continuum, while the tensor [J*_a_*] provides the derivatives related to rigid body rotations.

## 6. Differential Geometry Description of Deformation

This section gives the Eulerian description of the local transformations produced by the applied deformation of the RVE. The utilization of the Euler–Almansi tensor is recommended as a deformation tensor, since the recorded images provide the deformed configuration of the analyzed RVE.

**Figure 5:**
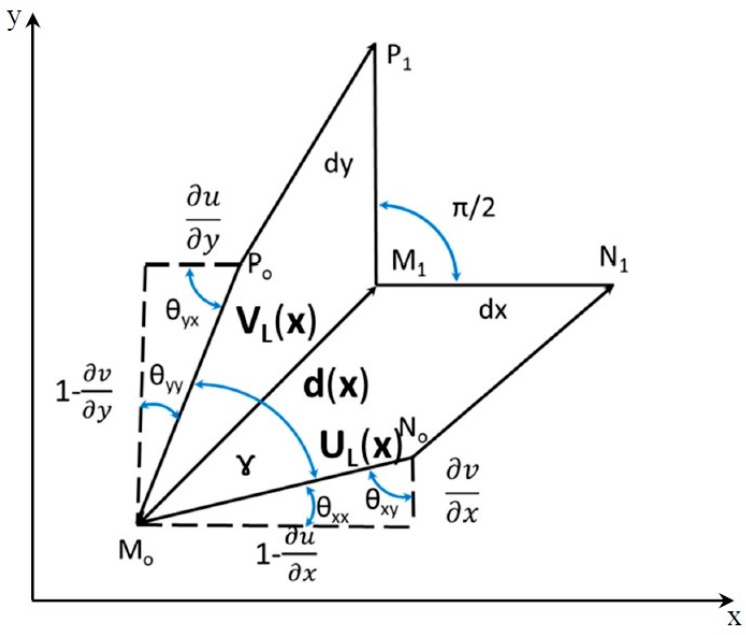
Differential geometry description of the local transformations entailed by the applied deformation to a continuum medium. The figure illustrates the 2D case.

Figure 5 illustrates the Eulerian description of the local transformations entailed by the applied deformation to a continuum medium. The Cartesian reference system represents both Lagrangian and Eulerian coordinate systems; both coordinate systems have parallel versors. The point M_o_ of the continuum displaces to M_1_, and the ***d***(***x***) vector joining these two positions is the same for both coordinate systems [9]. The two arc elements that after deformation become parallel to the Eulerian coordinate system correspond to the $U_{L}(x)={\bar{M_{o}N}}_{o}$ and $V_{L}(x)={\bar{M_{o}P}}_{o}$ vectors. It is necessary to realize that the elements of arc dx and dy that one measures in the deformed conditions are parallel to the axes of the Eulerian reference system.

The changes experienced by the medium (see Figure 5) are analyzed in terms of differential geometry. Hence, the full Euler–Almansi strain tensor whose components become 0 for a rigid body rotation is defined as:

(23) $$\left\{ \begin{array}{l} \varepsilon_{x}^{E}=1-\sqrt{1-2\frac{\partial u}{\partial x}+{\left(\frac{\partial u}{\partial x}\right)}^{2}+{\left(\frac{\partial v}{\partial x}\right)}^{2}}\\ \varepsilon_{y}^{E}=1-\sqrt{1-2\frac{\partial v}{\partial y}+{\left(\frac{\partial v}{\partial y}\right)}^{2}+{\left(\frac{\partial u}{\partial y}\right)}^{2}}\\ \left(\varepsilon_{xy}^{E}\right)=\arcsin\frac{\frac{\partial u}{\partial y}+\frac{\partial v}{\partial x}-\frac{\partial u}{\partial x}\frac{\partial u}{\partial y}-\frac{\partial v}{\partial x}\frac{\partial v}{\partial y}}{\left(1-\varepsilon_{x}^{E}\right)\left(1-\varepsilon_{y}^{E}\right)} \end{array}\right.$$

It should be noted that optical methods carry out experimental determinations with respect to deformed geometry. The linear theory (i.e., small deformation theory) assumes that θ_xx_ and θ_yy_ are small quantities. Furthermore, derivatives of displacements are much smaller than 1, and hence their squares and products also can be neglected. Thus, the difference between the initial and final geometries can be disregarded, and the initial geometry configuration can be used. Conversely, in the case of large deformations, the Lagrangian and the Eulerian descriptions lead to different results, and the latter is the most adequate approach in experimental mechanics, because it portrays the actual physics entailed by measurements.

There is another version of the Eulerian strain tensor, which is sometimes in the literature referred to also as the simplified Euler–Almansi tensor:

(24) $$\left\{ \begin{array}{l} \varepsilon_{x}^{E}=\frac{\partial u}{\partial x}-\frac{1}{2}\left[{\left(\frac{\partial u}{\partial x}\right)}^{2}+{\left(\frac{\partial v}{\partial x}\right)}^{2}\right]\\ \varepsilon_{y}^{E}=\frac{\partial v}{\partial y}-\frac{1}{2}\left[{\left(\frac{\partial u}{\partial y}\right)}^{2}+{\left(\frac{\partial v}{\partial y}\right)}^{2}\right]\\ \varepsilon_{xy}^{E}=\frac{1}{2}\left[\frac{\partial u}{\partial y}+\frac{\partial v}{\partial x}-\frac{\partial u}{\partial x}\frac{\partial u}{\partial y}-\frac{\partial v}{\partial x}\frac{\partial v}{\partial y}\right] \end{array}\right.$$

However, the above tensor is not equal to zero in the case of rigid body rotations. For example, for a rigid body rotation ϴ, $\varepsilon_{x}^{E}$ = sin^2^θ/2, which becomes almost equal to zero if θ is small. The simplified Euler–Almansi tensor hence represents a valid approximation in between linear theory where it is possible to neglect rotations and nonlinear theory entailing large rotations.

It has been pointed out in Section 5 that the symmetric tensor [J*_s_*] stated by Equation (21) is related to the deformation of the medium, while the anti-symmetric tensor [J*_a_*] stated by Equation (22) is related to local rigid body rotations of the considered volume element. It can be seen that the local rigid body rotation Ω(**x**) of a volume element is expressed as one-half of the curl of the local displacement field:

(25) $$\Omega =\frac{1}{2}\left|\begin{array}{ccc} i & j & k\\ \frac{\partial }{\partial x} & \frac{\partial }{\partial y} & 0\\ u & v & 0 \end{array}\right|=\mathrm{arctg}\frac{1}{2}\left(\frac{\partial v}{\partial x}-\frac{\partial u}{\partial y}\right)k$$

The difference of the cross-derivatives corresponds to the rigid body rotation of an element of area in 2D or, from the point of view of the 3D space, the rotation of an element of volume.

## 7. Relationships between Kinematical Variables and Dynamical Variables

The preceding sections introduced a mathematically consistent model for analyzing kinematical variables based on principles of continuum mechanics. These derivations match continuum mechanics theory with experimental mechanics observations. This section will focus on relating kinematical variables to dynamical variables. This step is needed both from the theoretical point of view and for assessing the accuracy of engineering applications.

As stated before, the representative volume element (RVE) is a bridge between the discontinuous nature of materials and continuum mechanics. Materials are characterized by their mechanical properties, and these properties represent averaged values at a certain subscale. Averages are computed at selected areas or volumes with a given shape that for convenience are squares in 2D and cubes in 3D. The required variables that apply to a given RVE are selected on the basis of the Hill–Mandel homogenization principle [5,6]. This selection involves the space of admissible displacements; it states that for certain σ_rve_ and ε_rve_, the virtual work in the macro scale equals the virtual work in the subscale. This relationship between kinematic variables and dynamic variables is of great practical significance, because the failure of fulfilling this principle can lead to considerable errors in the values of the computed quantities. This subject is covered in great detail in [23,24,25,26,27,28].

**Figure 6:**
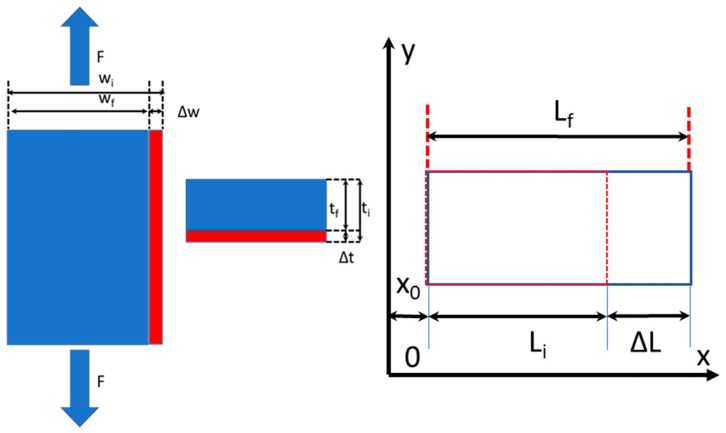
Tensile specimen subjected to uniform tensile stress, plane stress condition.

Let us start with an intuitive approach to relate kinematic and dynamic variables. Figure 6 illustrates the Eulerian description of a uniform displacement field developed in the continuum.

The longitudinal displacement of a generic point of the specimen can be expressed as:

(26) $$u(x)=\left(x-x_{0}\right)\frac{L_{f}-L_{i}}{L_{f}}$$

The differentiation of Equation (26) with respect to the x-coordinate yields:

(27) $$\frac{\partial u}{\partial x}=\frac{L_{f}-L_{i}}{L_{f}}=\varepsilon_{x}^{E}.$$

This strain corresponds to the value given by the Euler–Almansi strain tensor for a uniform 1D field (Equation (23)).

The strain tensor and the stress tensor depend on the selected description, Lagrangian or Eulerian. Since one is dealing with the deformed configuration, the strain is the Eulerian strain, the stress is the true stress, the force acts in the deformed configuration, and the corresponding stress tensor is the Cauchy stress tensor. Figure 6 shows that since the specimen is pulled in the axial direction, the cross-section changes and takes the instantaneous value A_f_ = w_f_ × t_f_. The Eulerian stress σ^E^ is equal to the ratio between the applied force and the actual cross-sectional area A_f_: that is, σ^E^ = F/A_f_.

**Figure 7:**
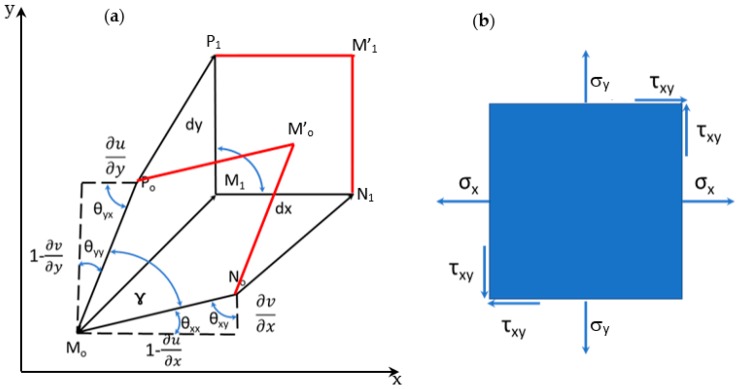
(**a**) The undeformed element M_o_N_o_M’_o_P_o_ becomes the square M_1_N_1_M’_1_P_1_ in the deformed position; (**b**) Stress components acting on an area element.

The basic derivation presented above can be extended to the general 2D case. Figure 7a shows that the M_o_N_o_M’_o_P_o_ parallelogram in the undeformed configuration deforms into the M_1_N_1_M’_1_P_1_ square. The components of the Euler–Almansi strain tensor, Equation (23), are evaluated for the M_1_N_1_M’_1_P_1_ square, which corresponds to the deformed position. As mentioned before, the stress is the true stress defined by the Cauchy stress tensor. Figure 7b shows the 2D true stress components corresponding to the deformed state. This relationship can be generalized for the six components of the Cauchy stress tensor in 3D. The arguments presented in this section hence prove that the Euler–Almansi strain tensor is compatible with the Cauchy stress tensor.

There is a deeper question to be answered: Are these two tensors conjugate in the Hill–Mandel sense? In the example that will be presented later on in the paper it is shown that, for hyperelastic materials, the obtained results satisfy both continuity conditions and equilibrium conditions. For metals experiencing large rotations and plastic deformations, the answer is more complex because it may depend on the molecular structure of the metal.

## 8. Derivation of Constitutive Models

To fully connect the kinematic and dynamic variables, it is necessary to review concepts in the field of constitutive models. This review is also necessary to help understand one of the examples of applications that are included in the paper.

The dynamics of the continuum is far more complex than the kinematics because it involves modeling the behavior of materials. Such a task may cover an extremely wide spectrum of cases. Constitutive models must be defined in order to relate the strain tensor with the stress tensor. Since the present study deals with the concept of the representative volume element of different materials, it is necessary to make some simplifications in the process of deriving constitutive models. First, the constitutive model should be path-independent in the RVE space. Second, in the selected RVE, the constitutive law should be time-independent. Third, physically valid constitutive models must not depend on the reference frame adopted in the modeling process. For example, Cauchy elastic materials satisfy the aforementioned conditions, but they represent just a mathematical abstraction. However, many engineering materials and biomaterials can be assumed to be Cauchy elastic materials in many applications of general interest.

The most general approach to the derivation of a constitutive model is to define an internal energy function containing some thermodynamic variables (e.g., temperature, entropy, etc.) as well as kinematic variables depending on the selected strain tensor. Furthermore, they include constant parameters or functions that must be experimentally determined. To perform the measurements, a model must be adopted that satisfies the compatibility conditions of strain and stress tensors. This fact also means that the strain energy function adopted in constitutive modeling depends on the adopted strain tensor and stress tensor. If internal energy W(**E**_ij_) is defined as a function of a given strain tensor **E**_ij_, and it is assumed to depend on the deformation gradient only, ignoring thermodynamic variables and energy dissipation processes, one can relate stress tensors and strain tensors by the equation

(28) $$\Sigma_{ij}=\frac{\partial W(E_{\mathrm{ij}})}{\partial E_{\mathrm{ij}}}.$$

The stress tensor **∑***_ij_* is the conjugate of the strain tensor in the definition of virtual work.

In the Eulerian description, **∑***_ij_* is the Cauchy stress tensor, while **E**_ij_ is the Euler–Almansi strain tensor. A simple extension of classical elastic mediums is the Saint Venant–Kirchhoff elastic medium, which is defined by a two-parameter energy function:

(29) $$W(E_{\mathrm{ij}})=\frac{C_{1E}}{2}{\left(\mathrm{tr}\left[E_{\mathrm{ij}}^{E}\right]\right)}^{2}+C_{2E}{\left(\mathrm{tr}\left[E_{ij}^{E}\right]\right)}^{2}$$

where C_1E_ and C_2E_ are elastic parameters corresponding to the Lamé constants of linear elasticity.

Differentiating the W function with respect to **E_ij_**, the stress tensor **∑*_ij_*** is obtained from Equations (28) and (29):

(30) $$\Sigma_{\mathrm{ij}}=\frac{\partial {W(E}_{ij}^{E})}{\partial E_{ij}^{E}}=C_{1E}\mathrm{tr}(E_{ij}^{E})+2C_{2E}E_{ij}^{E}.$$

The strain tensor to be introduced in Equation (30) is the Euler–Almansi strain tensor, while the stress tensor obtained as the output from Equation (30) is the Cauchy stress tensor. Hence, it follows:

(31) $$\Sigma_{ij}=\left[\begin{array}{cc} \sigma_{x} & \tau_{\mathrm{xy}}\\ \tau_{\mathrm{xy}} & \sigma_{y} \end{array}\right].$$

Using Equation (30), the components of the Cauchy stress tensor, Equation (31), are expressed as:

(32) $$\{\begin{array}{l} \sigma_{x}=C_{1E}\left(\varepsilon_{x}^{E}+\varepsilon_{y}^{E}\right)+2C_{2E}\varepsilon_{x}^{E}\\ \sigma_{y}=C_{1E}\left(\varepsilon_{x}^{E}+\varepsilon_{y}^{E}\right)+2C_{2E}\varepsilon_{y}^{E}\\ \tau_{\mathrm{xy}}=2C_{2E}\varepsilon_{\mathrm{xy}}^{E} \end{array}.$$

## 9. RVE and Experimental Mechanics Measurements

In the Introduction section, it is stated that a main goal of the paper is to verify continuum mechanics theoretical predictions utilizing results obtained from experimental mechanics. Since the concept of RVE is the bridge between continuum mechanics and the discontinuous structure of actual materials, to achieve this goal, it is necessary to connect the abstract concept of RVE and experimental measurements. For this purpose, an example is selected that sharply shows the importance of the RVE in experimental measurements. A fundamental variable in the adoption of a RVE is the selection of the scale of the RVE. The determination of local kinematic variables for a selected RVE in the general case of large deformations and rotations requires removing limitations due to the use of the linearized kinematics. Displacement fields are no longer described in a unique reference system; the difference between the undeformed and deformed shapes cannot be neglected.

**Figure 8:**
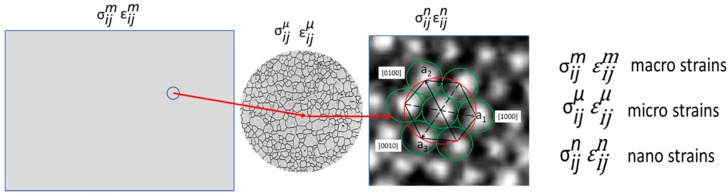
Illustration of three scales of experimental observation of images captured in experimental mechanics.

One has to select a given RVE scale and the corresponding coordinate system to define local variables, choosing either a Lagrangian or an Eulerian representation. Figure 8 conveys graphically the concept of measurement of mechanical properties at three different scales. Actually, there are many different scales that can be introduced; each one will provide different aspects of the kinematics of the observed materials and by selecting suitable constitutive functions one can get stress distributions at different scales. The observed fields are scale dependent and also depend on the spatial resolution that can be achieved. The same field observed with different spatial resolution will provide different results depending on the gradients of the selected variables in the field of interest.

The following relationships between scales should be valid:

(33) $$\sigma_{ij}^{m}\left(x\right)=<\sigma_{ij}^{\mu }\left(x\right)>=\frac{1}{V_{R}}\underset{V_{R}}{\iint }\sigma_{ij}^{\mu }\left(x_{R}\right){\mathrm{dV}}_{R}$$

(34) $$\varepsilon_{ij}^{m}\left(x\right)=<\varepsilon_{ij}^{\mu }\left(x\right)>=\frac{1}{V_{R}}\underset{V_{R}}{\iint }\varepsilon_{ij}^{\mu }\left(x_{R}\right){\mathrm{dV}}_{R}.$$

The meaning of Equations (33) and (34) is that the field average at a lower scale, as shown in Figure 8, should provide the values of the stresses and strains of the corresponding point of the upper scale. There are other important requirements that need to be satisfied; it is necessary to adopt a strain tensor that satisfies the condition of invariance of the strains upon rigid body rotations. Concurrently, it is necessary to adopt a stress tensor that is compatible with the selected strain tensor. In experimental mechanics determinations, since images are obtained in the deformed state, it is more convenient to work with the Eulerian description.

**Figure 9:**
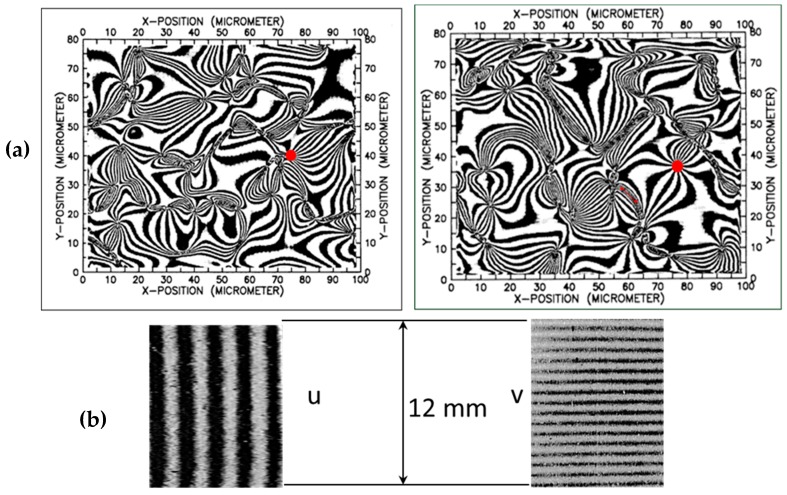
(**a**) Patterns of the *u* and *v* displacements of the Al–SiC tensile specimen in a region of size 100 × 80 μm^2^ (micron scale); (**b**) Patterns of a region of 12 mm of the tensile specimen (mm scale).

Microscopic patterns corresponding to a metallic particulate composite, Al–SiC [14,29], illustrate the scale transitions in the case of complex fringe patterns. The images of the particulate composite contain a variety of singularities that are difficult to analyze utilizing traditional techniques of pattern unwrapping. The four images of Figure 9 correspond to a tensile specimen observed at two different scales, the millimeter scale and the micron scale. The patterns at each scale are quite different but are connected through Equations (33) and (34).

The moiré optical setup used in the experimental investigations of the Al–SiC composite specimen is described in detail in Refs. [14,29]. A microscope with axial illumination and viewing observes the tensile specimen illuminated by white light. A CCD camera records patterns in real time as the specimen is loaded. A cross-grating of pitch p = 833 nm was engraved in the tensile specimen using photo-resist. Interpolation algorithms reduced the pitch of the printed grating from p = 833 nm to p = 55.6 nm: this allowed obtaining the moiré pattern at the micron scale.

The Al–SiC specimen was tested using a servohydraulic Instron machine. The tensile load was supplied to the specimen in a quasi-static fashion following the directions of ASTM standards on the tensile testing of composite materials. The optical setup was rigidly connected to the testing machine in order to minimize vibrations and parasitic movements. The tensile test was replicated on several specimens with the same geometry and dimensions in order to obtain statistically significant results.

**Figure 10:**
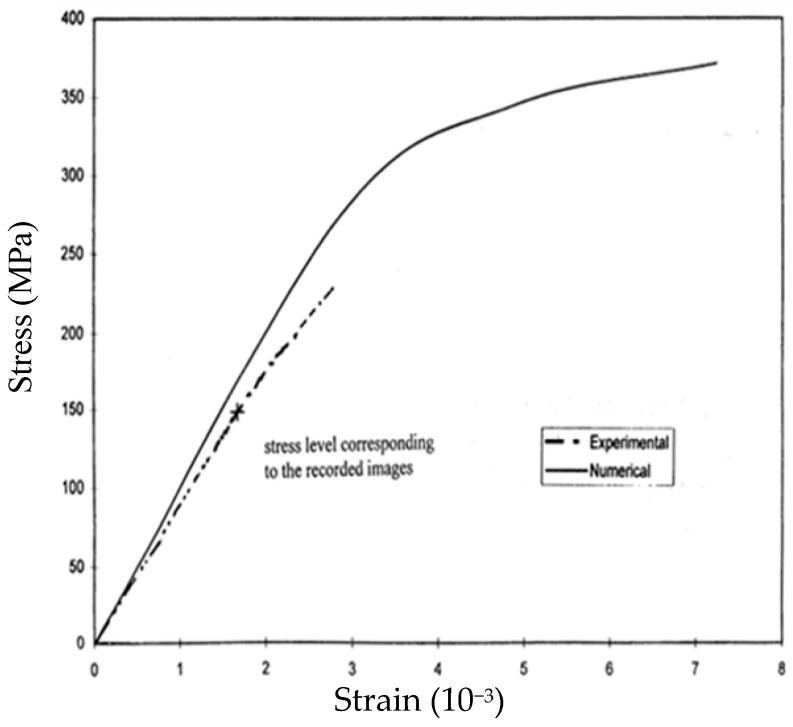
Stress–strain curves of the Al–SiC composite specimen subject to tensile loading.

Each Al–SiC tensile specimen was cut from a plate of Al 536 material reinforced with silicon carbide (SiC) particles fabricated at the IIT-IITRI (Illinois Institute of Technology and Illinois Institute of Technology Research Institute, Chicago, IL, USA) facilities; the volume fraction of SiC reinforcement is 20%. The composite material was built by mixing the Al 536 and SiC components at high temperature. The technological process leading to the formation of the composite material is well documented in the classified reports of the US Airforce division involved in the research. For the linear elastic regime, a classical mixture rule was used for determining the mechanical properties of the Al–SiC composite based on the properties and volume fractions of each constituent of the mixture. These properties were confirmed by the experimental tests. Then, a detailed finite element (FE) study [30] of the composite was performed, and the corresponding stress–strain curve was computed in the elasto-plastic regime and compared with experimental data (see Figure 10).

The *u* and *v*-displacement patterns presented in Figure 9 correspond to the composite stress σ = 149.38 MPa and strain ε_y_ = 1.675^.^10^−3^ (i.e., 1675 με). These values are related by the stress–strain curve shown in Figure 10. It is necessary to point out that the observed singularities of the patterns correspond to projected displacements. Most of the singularities are not physical discontinuities but correspond to discontinuities of the projected displacement field fringes [7]. In Figure 9a, there are two areas indicated in red that, due of the high spatial resolution of the images, could be studied at the nanometer scale. The detailed results of this analysis are presented in [14].

Figure 10 shows two stress–strain curves of the tensile specimen: one curve corresponds to the experimental stresses and strains of the measurements performed in the tensile test, and the other curve corresponds to the stresses and strains given by the FE computations [30]. For low stresses, the FE results accurately match the tensile test results; as stresses increase, a more significant reduction of the modulus of elasticity is observed. The reduction of E means an increasing damage of the composite. A brittle rupture of the composite takes place at a load well below the fracture predicted by the J_2_ flow rule utilized in the FE computations [30].

Figure 10 shows that up to a certain level of stress, the Al–SiC composite behaves as a quasi-elastic material. From the point of view of the macropatterns shown in Figure 9b, the fringes correspond to a tensile specimen. The frequency of the pattern is higher in the y-direction (V displacement pattern) along which the tensile load is applied to the specimen. The lower spatial frequency of the moiré pattern in the x-direction (U displacement pattern) indicates a contraction of the specimen in the orthogonal direction to the applied load, and this deformation is ν_c_ times the elongation experienced by the specimen in the y-direction. In the micron scale, the field of displacements is extremely complex; Figure 9a shows very high strains and rotations.

Table 1 shows the composition of the analyzed material and the modulus E and Poisson’s ratio ν_c_ obtained from the FE analysis [30]. The values in the table are consistent with the values reported in the literature for the 20% volume content of the SiC. It was concluded that the cause of the brittle behavior of the composite and the early fracture is due to the irregular shapes of SiC particles and their irregular distribution. These two factors produced high strain gradients [29] that inhibited the motions of the dislocation that adapt the material to the applied deformations in the plastic behavior of the material implicit in the J_2_ flow rule [30].

**Figure 11:**
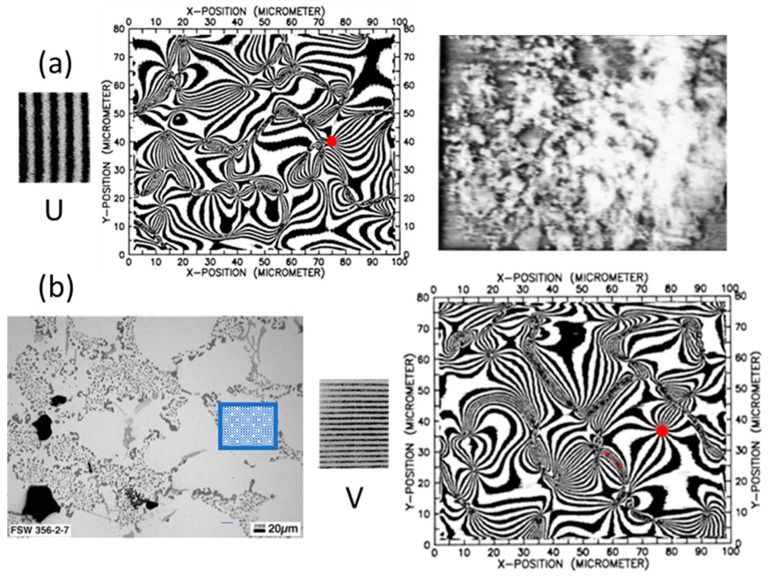
(**a**) Moiré U and V patterns of an 100 × 80 μm^2^ area of a tensile Al–SiC composite specimen with an aluminum matrix reinforced by SiC particles. Corresponding U and V patterns in the mm scale and microphotography of the observed region, black areas SiC particles, bright areas aluminum matrix; (**b**) Metallographic image of the aluminum matrix and the observed region of 100 × 80 μm^2^. The observed region is smaller than the average grain size. Reprinted from [14] with permission from Elsevier.

The analysis of the behavior of the material at different scales provided clues concerning a very important technological process: the fabrication of particle-reinforced metal composites. As shown in Figure 11, the images of the areas of 100 × 80 μm^2^ of Figure 9a correspond to a region smaller than the average grain size of the matrix. Figure 11 also shows a heterogeneous distribution of SiC particles of irregular shapes.

**Figure 12:**
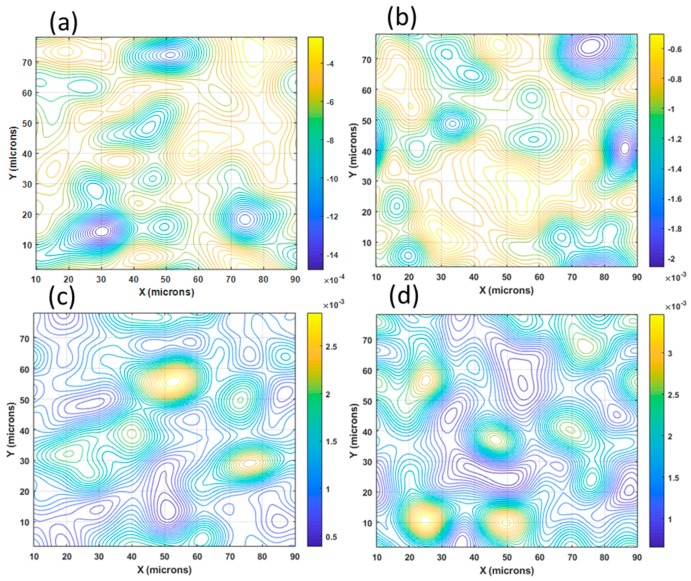
Derivatives of projected displacements determined for the tensile Al–SiC composite specimen: (**a**) ∂u/∂x; (**b**) ∂u/∂y; (**c**) ∂v/∂x; and (**d**) ∂v/∂y.

**Figure 13:**
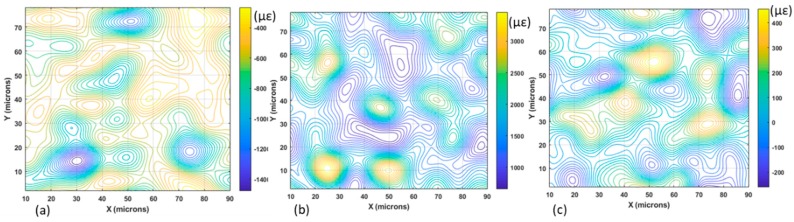
Components of the full Euler–Almansi strain tensor determined for the tensile Al–SiC composite specimen: (**a**) ε_x_^E^; (**b**) ε_y_^E^; and (**c**) ε_xy_^E^.

In order to verify the validity of Equation (34), the derivatives of the displacement patterns of Figure 9a are computed, as shown in Figure 12a–d. These derivatives are processed with the HoloStrain software [31] using the phase-to-strain function. The computed values are smoothed using a filter in order to limit the peak values that correspond to the presence of fringe patterns dislocations [7] (i.e., areas of fringes discontinuities). Then, these derivatives are given as input to Equation (23) in order to determine the full Euler–Almansi strain tensor. The corresponding maps of the strain components are shown in Figure 13a–c.

Both the derivatives and the strains are numbers, but the strain values are multiplied by 10^6^. The derivatives of the displacements ∂u/∂x (Figure 12d) and ∂v/∂y (Figure 12d), that in the small-deformation theory are ε_x_ and ε_y_, are different from ε_x_^E^ (Figure 13a) and ε_y_^E^ (Figure 13b), which provide the true strains of the field, but they have the same order of magnitude. The 10^6^ multiplication factor shows that the microstrain values given in Figure 13 have the same order of magnitude as the derivative values given in Figure 12.

**Figure 14:**
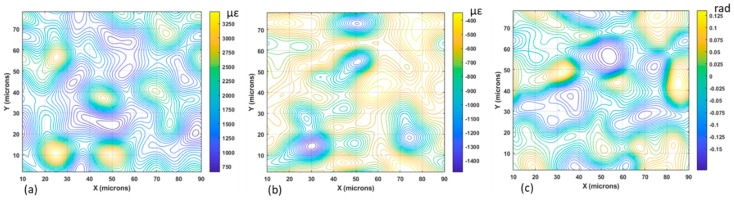
Al–SiC composite specimen: (**a**) Principal strain ε_p1_; (**b**) Principal strain ε_p2_; (**c**) Angle defining the direction of principal strains θ_p_.

**Figure 15:**
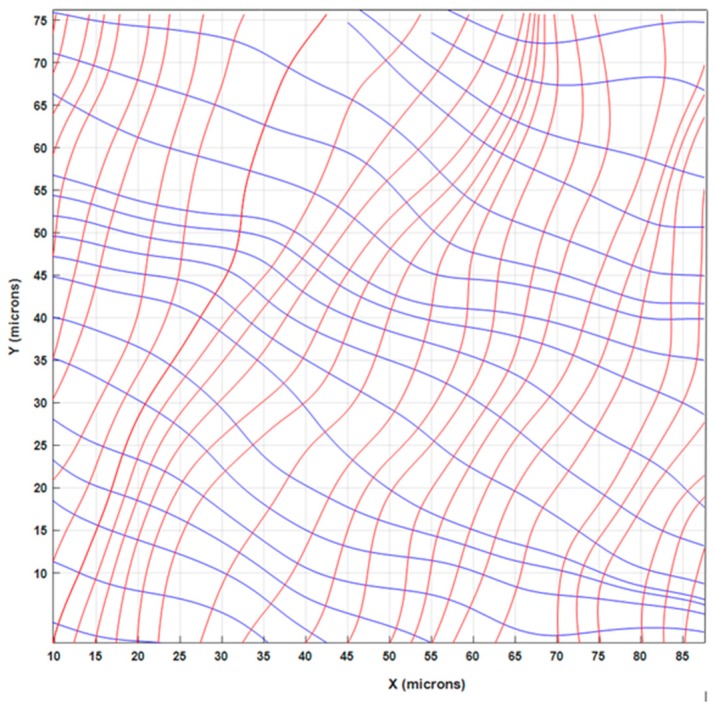
Trajectories of the principal strains in the representative volume element (RVE) of the Al–SiC composite specimen.

Figure 14a,b show the maps of principal strains, while Figure 14c shows the corresponding angles defining the direction of principal strains. The trajectories of principal strains are represented in Figure 15.

The analysis of the behavior of the material at different scales provided clues concerning a very important technological process: the fabrication of particle-reinforced metal composites.

Table 2 contains the information corresponding to the verification of Equation (34) for the Al–SiC composite specimen. As mentioned above, the moiré patterns of the pseudo-homogeneous material (bottom part of Figure 9a) correspond to a measured strain ε_y_ of 1675 με at the macro scale and a tensile stress of 149.4 MPa; these values are reported in the first two columns of Table 2. The third column of the table reports the value of the secant elastic modulus E_c_, which is the quotient of columns 1 and 2. The fourth column of the table reports the average value of the true strain in the y-direction ε_y_^E^ obtained by processing the microscopic patterns of Figure 9a. The fifth column of the table reports the average value of true strain in the x-direction ε_x_^E^, which was obtained via image processing. The sixth column of the table reports the absolute value of ratio between the strain values listed in the fifth column and the fourth column, which represents an equivalent Poisson’s ratio.

The last column of Table 1 is the percent difference between the values reported in the second and fourth columns. The two values of ε_y_, the one coming from the stress–strain curve obtained at the macro scale and the average of ε_y_^E^ obtained by processing the microscopic moiré patterns match very closely; there is only a 1.4% difference. These results verify the validity of Equation (34). Hence, the selected region of about 100 × 80 μm^2^ is the RVE size, ensuring the convergence of local strains to the strains of the pseudo-homogeneous material within a certain number of significant figures.

Interestingly, the strain value ε_y_^E,simpl^ determined by averaging the local strain field, which has been obtained by giving displacement derivatives in the input to the simplified Euler–Almansi tensor of Equation (24), does not match the measured strain at the macro scale. In fact, it is only 1238 με versus 1675 με, with a percent error of 26.1%. This result confirms the deep connection between the selection of a certain RVE and the kinematics of deformation assumed for that RVE. Here, the presence of SiC particles alters by a great extent the strain field at the interface with the Al matrix, and this requires the correct modeling of kinematical behavior besides the selection of a proper RVE size. The concourse of these two factors was properly assessed in this study, thus allowing accurately matching the local strain field with the macro-scale observations.

The measured strain at fracture for the Al–SiC composite specimen is ε_u_ = 2790 με, while the microscopic patterns of Figure 9a correspond to a strain level of 1675 με. Hence, strain concentrations near singularities cause the fracture of the matrix already at 66% of the ultimate strain of the composite. The elasticity modulus drops from the 99 MPa value recorded at the origin to the value of 89.2 MPa corresponding to the recorded patterns. Hence, local damage resulted in a 10% reduction of the elastic modulus. The values of Poisson’s ratio obtained from FE analysis and the image processing analysis change from 0.33 to 0.39: such an 18.2% difference indicates a process of increment of the Poisson’s ratio in coincidence with the increasing plastic deformation of the specimen. 

The principal strains in Figure 15 have opposite signs over the whole processed image, which is an effect of the tensile axial loading, while the transversal strains are due to the Poisson effect. This configuration of the isostatic lines corresponds to the fringe patterns of Figure 9b that at the mm scale have the same shape as that of a tensile specimen in the elastic range. 

The analysis presented in this section indicates that care should be taken in selecting the RVE for a heterogeneous material. While it is obvious that the selected representative volume element must provide reliable information on the actual behavior of the analyzed material, it should be underlined that same ensemble averages may correspond to very different local behaviors. This is especially true in the present case, because the high heterogeneity of the investigated composite material deriving from the irregularity of the SiC particle shapes resulted in highly variable strain distributions at the interface between the Al matrix and each SiC particle. Remarkably, the RVE selected for the analysis of the Al–SiC composite proved to be very effective. In fact, besides the convergence of local strain values to the average values measured at the macro scale and the consistency of Poisson’s ratio values (this was achieved by properly selecting the kinematics of deformation followed by the RVE), concentrations of very high strains are localized, and they reduce the global stiffness of the composite by only 10%, although local damage in the aluminum matrix already occurred at 66% of the ultimate strain recorded in the experimental investigation. Inside RVE, the ε_y_^E^ strains may raise up to about 3250 με (see Figure 13), which is almost twice the average value of 1651 με, but these peaks cannot not cause a generalized failure of the material.

## 10. Experimental Verification of Constitutive Models

The validity of the constitutive equations of the RVE derived in Section 8 is verified by utilizing the strain and stress distributions in an urethane rubber disk under diametrical compression [32]. The specimen underwent moderately large deformations. The diameter of the disk is 4 inches (10.16 cm), while the thickness t is 0.5 inches (1.27 cm). The initial cross-sectional area is A_o_ = 4 × 0.5 = 2 in^2^. Experimental measurements were performed with a classical intrinsic moiré setup using white light illumination. A 300 lines/in grating (pitch = 84.67 μm) was photo-engraved in the disk. Two standard tensile specimens with a cross-sectional area of 0.629 in^2^ were considered as well. The disk and the tensile specimens were all machined from the same cast plate of material. The same grating was photo-engraved in the longitudinal direction in one tensile specimen and in the transversal direction in the other tensile specimen.

**Figure 16:**
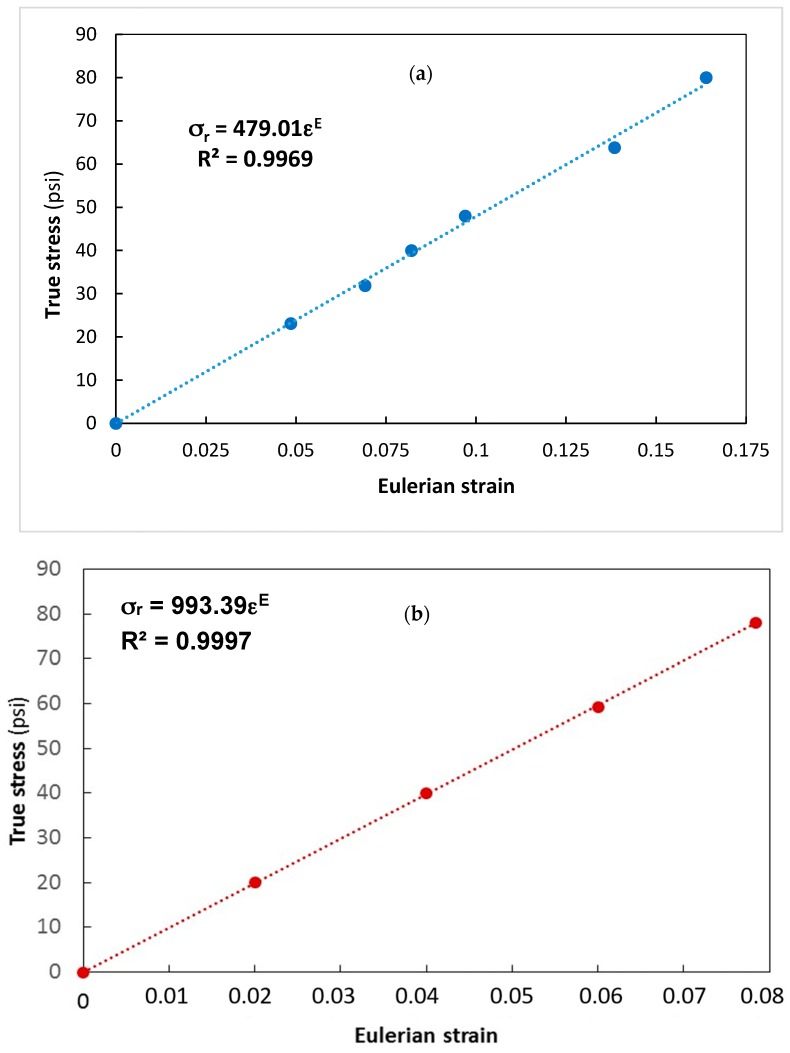
Results of the tensile tests utilized to determine: (**a**) elastic constant C_1E_; (**b**) elastic constant C_2E_ of urethane rubber.

Figure 16a shows the experimentally measured true strains and true stresses for the first tensile specimen, while Figure 16b shows the corresponding values obtained for the second tensile specimen. Experimental data are very well fitted by linear regression: in fact, the values of the correlation coefficient R^2^ are 0.9969 and 0.999. The slopes of the fitting lines correspond to elastic constants. In particular, it can be seen from Figure 16a that C_1E_ = 479.01 psi (C_1E_ = 3.304 MPa), while Figure 16b shows that C_2E_ = 993.39 psi (C_2E_ = 6.852 MPa). The ratio of the two elastic constants is r_c_ = 0.482; this figure is very close to the condition r_c_ = 0.5, which is valid for an incompressible material.

The elastic constants C_1E_ and C_2E_ thus determined were given as input to the constitutive Equation (32) in order to compute the stresses developed in the disk under diametrical compression.

**Figure 17:**
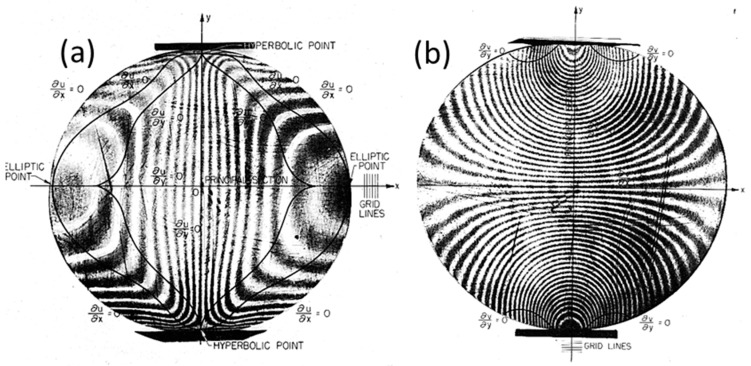
Moiré fringes generated for the urethane rubber disk subject to diametrical compression: (**a**) *u*-displacement pattern; (**b**) *v*-displacement pattern. Signs of displacement derivatives, lines where displacement derivatives are equal to zero, and singular points are indicated in the figure as well.

Figure 17 shows the moiré patterns obtained for the diametrically compressed urethane rubber disk. The y-direction corresponds to the direction of the applied load. The grating lines used in the experiments also are indicated in the figure: the vertical lines are sensitive to the horizontal displacements *u* (Figure 17a), while the horizontal lines are sensitive to the vertical displacements *v* (Figure 17b). The moiré fringes were generated by superposing the images recorded for the undeformed and the deformed configurations. Figure 17 indicates the signs of displacement derivatives as well as the lines where displacement derivatives are equal to zero and the singular points where the fringe slope is indeterminate. Moiré patterns were processed in the Fourier space using carrier modulation and fringe extension techniques. More details on the image processing methods utilized for this example are given in [7].

**Figure 18:**
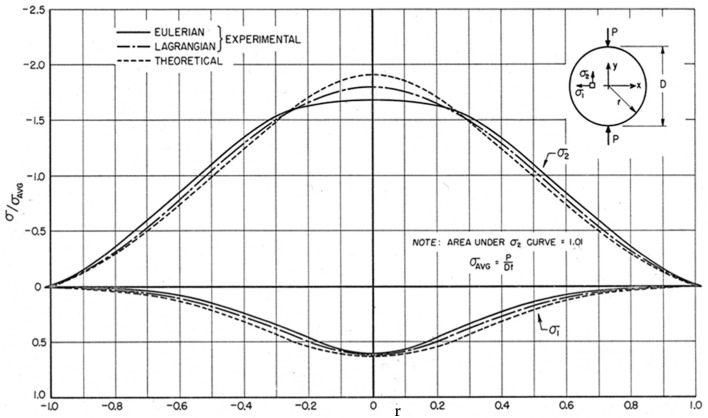
Comparison of experimental and theoretical distributions of principal stresses along the disk horizontal diameter.

The unloaded disk has a circular shape, while the deformed specimen becomes quasi-elliptical under the action of the applied load. The y-axis remains an axis of symmetry for the deformed configuration. Figure 18 shows the distributions of principal stresses σ_1_ and σ_2_ developed along the horizontal diameter of the disk. A dimensionless representation is utilized to better compare the different results presented in the figure: the horizontal axis of the plot reports r, while the x-coordinate divided by the radius of the deformed disk and the points limiting the horizontal diameter are denoted by r = ±1.

Stress values were computed by inputting the measured Eulerian strain values to the constitutive Equation (32). Then, principal stresses were computed, and the corresponding values were finally normalized with respect to average stress σ_avg_. The average stress was computed via the numerical integration of stress plots. The “theoretical” stress curves shown in Figure 18 correspond to the classical elasticity solution of the diametrically compressed disk [33].

Along the horizontal diameter there are no rotations; the strains are principal strains, and the derivatives of the displacements directly give the strains. Hence, the Lagrangian strain $\varepsilon_{x}^{L}$ in the horizontal direction can be obtained from the measured Eulerian strain $\varepsilon_{x}^{E}$ as follows:

(35) $$\varepsilon_{x}^{L}=\frac{\varepsilon_{x}^{E}}{1-\varepsilon_{x}^{E}}.$$

The obtained strains can be utilized to get the stresses. The average of the dimensionless Eulerian stress curve was 1.01, indicating an error of 1% in the condition of the static vertical equilibrium.

**Figure 19:**
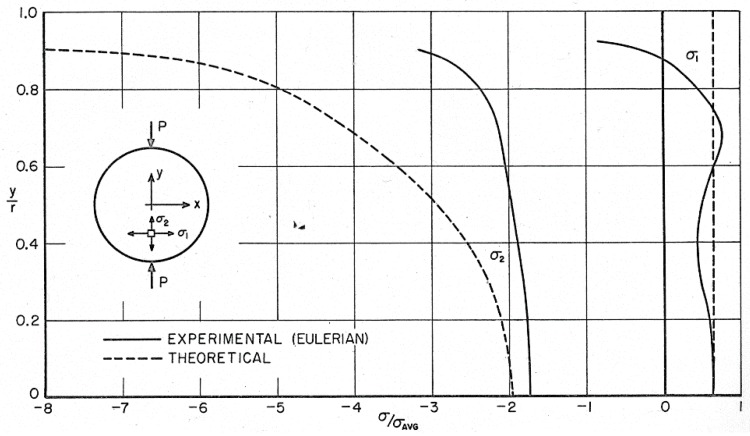
Comparison of experimental and theoretical distributions of principal stresses along the disk vertical axis of symmetry.

Figure 19 shows the distributions of principal stresses along the vertical symmetry axis of the disk (i.e., the y-axis). The experimentally determined stress σ_2_ differs considerably from the theoretical solution [33], which assumes a concentrated force (i.e., point force singularity), while the real load acting on the disk is the resultant of contact stresses developed at the interface between the specimen and the steel bar that transfers the load to the disk. The stress σ_1_ becomes negative near the contact region.

**Figure 20:**
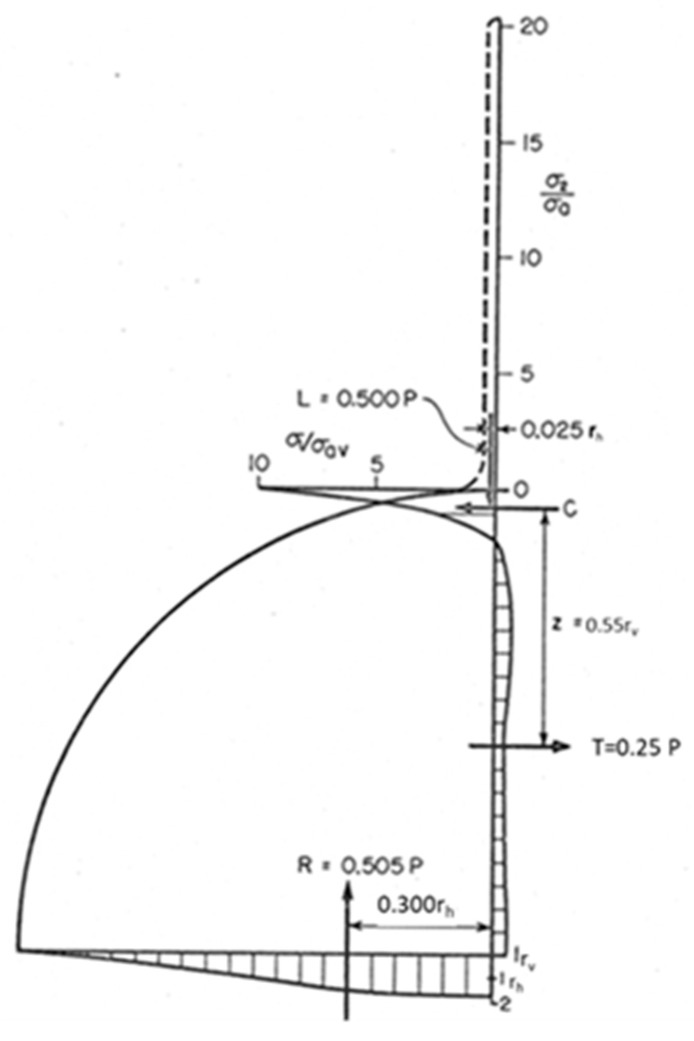
Moment equilibrium conditions obtained from stress distribution in one quarter of the disk.

In order to verify that the Euler–Almansi strain tensor and the Cauchy stress tensor are compatible, the RVE stresses derived from the constitutive Equation (32) must satisfy the conditions of static equilibrium. Due to the symmetry of σ_1_ stress patterns with respect to the y-axis, the condition of the zero resultant force is satisfied in the horizontal direction. In the vertical direction at different depths, the integrated curves of σ_2_ satisfy equilibrium within a 2% error. Stress distribution near the contact region between the disk and the loading bar was analyzed in detail by adopting a larger scale. Figure 20 shows that at the depth of 0.10r_h_, there is a point where σ_x_ = 0. Furthermore, the σ_x_ stress turns compressive in a contact region sector with an approximate width of 0.075r_h_. In a smaller region, about 0.05r_h_ wide, vertical stresses σ_y_ are distributed with a lever arm of about 0.025r_h_. In Figure 20, the deformed disk is approximately an ellipse of a major axis r_h_ and minor axis r_v_.

In the equilibrium equation written for one-quarter of the disk, positive moments are directed clockwise. The vertical forces involved in the equilibrium equation are the reaction force R, the load P applied to the disk, and L = −0.500P, corresponding to one-half of the contact stress resultant. The dimensionless coordinates (r_h_,r_v_) at any point of the disk are normalized with respect to the length of the major axis and the length of the minor axis of the deformed disk, respectively. With reference to Figure 20, the moment of the vertical forces M_v_ is:

M_v_ = 0.505P × 0.3r_h_ − 0.025r_h_ × 0.5P = 0.139Pr_h_. (36)

Since the equilibrium of horizontal forces involves that C = −T = 0.25P, their resultant moment M_h_ is:

M_h_ = −0.55r_v_ × 0.25P = −0.1375Pr_v_. (37)

The sum of the moments ∑_M_ is

∑_M_ = M_h_ + M_v_ = 0.139P × r_h_ − 0.1375P × r_v_. (38)

The relationship between r_v_ and r_h_ is

r_v_ = 0.97r_h_. (39)

Replacing Equation (39) in Equation (38) yields

∑_M_ = 0.0067Pr_h_. (40)

Since ∑_M_ became zero to approximately two significant figures, the 0.0067Pr_h_ residual error in the moments is approximately 1%. From the above derivations, the equilibrium conditions are satisfied, since the continuity conditions are also satisfied. Hence, we have a solution of the disk under diametrical compression in the Eulerian description and in range of the applied deformations. Within a small experimental error, the Euler–Almansi strain tensor and Cauchy stress tensor are hence proven to be conjugate in the definition of virtual work.

## 11. Discussion and Conclusions

The general goal of the study is to connect theoretical predictions of continuum mechanics with actual experimental observations that support these predictions. In this process, the concept of the representative volume element (RVE) is an essential point. Experimental observations depend on the scale of the regions of interest, the RVE, and all the developments that are related to the properties of adopted RVEs at different scales.

In order to achieve the aforementioned goal, the paper reviews the kinematics of large deformations and large rotations in 2D in the context of isothetic lines (moiré fringes) applying recent original work of the authors in this field. The conversion of scalar quantities of gray-level images recorded by a light sensor into vectorial fields is analyzed. The original work of the authors provides the connection of scalar potential functions and their representation in the complex plane, making it possible through the corresponding software to compute the necessary kinematic variables. The next step is to review the properties of the derivatives of the displacements, the main tools in the implementation of measures of deformations that comply with a fundamental property like the invariance of these measures with rigid body rotations in the RVE.

By describing the local behavior of a deformed continuum in terms of differential geometry, it is possible to derive the Euler–Almansi strain tensor, which has useful properties in the description of the deformed continuum. The kinematics of the continuum must be related to the continuum dynamic variables; constitutive functions are discussed in the paper. The Hill–Mandel condition is an essential element of the process of connecting dynamic and kinematic variables in the RVE. In the paper, we developed an intuitive and novel connection between the Euler–Almansi strain tensor and the Cauchy stress tensor, pointing out that this conjugate compatibility still needs a validation in the Hill–Mandel sense. The Saint Venant–Kirchhoff elastic medium is chosen in this paper in order to illustrate the connection of the Euler–Almansi strain tensor and the Cauchy stress tensor.

The theoretical derivations of the paper are illustrated with two examples of application. The connection between different scales is illustrated with a very interesting and highly illustrative example of the study of a metal matrix reinforced by hard particles. This example connects a number of publications of the authors resulting from a very comprehensive study on the properties of an aluminum matrix reinforced with SiC particles; the study was supported by a USAF grant. The second example deals with a very useful model for a wide variety of actual materials: the Saint Venant–Kirchhoff elastic medium.

An alternative to the RVE is the statistical volume element (SVE), which is referred to in finite element theory as the stochastic volume element. While RVE utilizes statistical averages, SVE deals directly with solutions implementing the theory of continuum random fields.

In the application of these disciplines toward achieving safe designs, experimental mechanics plays a twofold fundamental role: (i) to verify predictions of theoretical and computational models; and (ii) to propose new ideas and generate new concepts for creating models that can give practical answers to many engineering applications. This study gives some partial answers to some of these questions utilizing the RVE concept and bringing some insights to the role of experimental mechanics in understanding why things fail and break.

## Figures and Tables

**Table 1:** Properties of the Al–SiC particle-reinforced composite.

Matrix	Reinforcement	Size (μm)	Vol %	E (GPa)	ν_c_
Al 536 Aluminum	SiC	10–20	20	99	0.33

**Table 2:** Summary of the main information regarding the Al–SiC particulate composite test.

σ Applied Stress in the Test	ε_y_ Measured Strain at Macro Scale	E_c_ Secant Mod.	ε_y_^E^_,avg_ Field Avg. y-strain	ε_x_^E^_,avg_ Field Avg. x-strain	ν_c_ Poisson’s Ratio	Percent Error on ε_y_
149.4 MPa	1675 με	89.2 MPa	1651 με	−645 με	0.39031	1.4%
